# Using the experiences of COVID to reposition services for children and adolescents with anorexia nervosa

**DOI:** 10.1177/10398562231174693

**Published:** 2023-05-10

**Authors:** Belinda Caldwell, Salvatore Catania, Matt Farrugia, Maria Ganci, Martin Pradel, Julia Quin, Alicia Tompson, Tania Withington, Tracey D Wade

**Affiliations:** Abbotsford, NSW; South Brisbane, QLD; Parkville, VIC; Parkville, VIC; Parkville, VIC; Abbotsford, NSW; Parkville, VIC; South Brisbane, QLD Brisbane, QLD; Bedford Park, SA

Dear Editor,

*The impact of the pandemic on eating disorders*: The COVID-19 pandemic, associated with a 15.3% increase of overall incidence of eating disorders, corresponded to an increased demand for eating disorder services in Australia. This resulted in several paediatric services revisiting the way they delivered timely and suitable interventions for anorexia nervosa. This letter describes four such responses, only one of which has been formally evaluated.^1^ We also summarise important principles from service providers across Australia to whom these service delivery models were described at a workshop.

*Innovative service delivery in challenging times*: The themes of the four different service models, described in Table 1, were (1) increase accessibility via hybrid service delivery; (2) increase timeliness of service response by introducing a brief intervention to increase carer empowerment to action; (3) consistent educational components of interventions including parent knowledge and skills, re-nourishment and refeeding, distress tolerance and communication; (4) incorporation of guided or group intervention; (5) interventions informed by current evidence; (6) utilising lived experience across design, delivery and evaluation; (7) evaluation of outcomes.

**Table 1.** Descriptions of, and comparisons between, the four online interventions.

Intervention	Intended Audience	Content	Support Provided	Outcomes	Challenges
Guided self-help family-based treatment (GSH-FBT) for families on the waitlist for treatment of AN^1^	Parent(s)/carer(s) whose child had a diagnosis of AN/atypical AN between the ages of 12 and 18 years and was medically stable, and who were willing to participate in weekly medical monitoring and on a waitlist to receive treatment with one of three specialist ED services in WA and SA	12 online modules accessed weekly; each included short videos with a clinician outlining FBT principles, an assigned reading from the book ‘Help your Teenager Beat an Eating Disorder’ and homework asking carers to track the use of weight in the child	Weekly zoom support by therapist guides for up to 30 min; postgraduate clinical psychology trainee guides (training and weekly supervision provided). Guidance limited to helping parents apply the knowledge learned	Large effect size improvements (Cohen’s d: 1.11–2.61) in carer knowledge, child ED behaviours and BMI centile/weight (mean increase of 5.89 kg over 12 weeks)	Poor feasibility, only 13% of eligible families expressed an interest in participating: initial rapid face-to-face assessment and discussion of study achieved best recruitment
Lived-experience carer-led peer support and psychoeducation for carers with the aim to provide early intervention to enable families to refeed their YP	Parent(s)/carer(s) whose child has a recent diagnosis of a restrictive eating disorder (e.g. AN, AAN, BN, ARFID). Child aged 19 or under. Waitlisted for clinical treatment (i.e. FBT). Being medically monitored weekly	8 structured carer coaching sessions via Zoom, on a range of specific topics relating to eating disorders, family led refeeding, distress tolerance and self-care. Extensive resources emailed at the conclusion of each coaching session	Weekly Zoom support by lived-experience carer coaches. Each session up to 1 h, 1:1 with a carer coach. Carer coaches receive clinician eating disorder training, FBT training and regular supervision	90 families completed part/all the program; 80% chose to complete all 8 sessions. Feedback overwhelmingly positive with participants reporting feeling informed, contained, supported and empowered to begin refeeding with the support of carer coach	Promotion of the program remains challenging and a dedicated promotion and/or advertising campaign to the CAMHS/CYMHS services could assist with recruitment of families meeting criteria
Treatment preparation clinic to: medically monitor YP; maintain engagement with families on waitlist; provide parents/carers with information and skills to support YP waiting for treatment; potentially reduce overall treatment time	Families where YP has been assessed as eligible for the program but awaiting treatment availability. YP are seen in the Specialist Medical Review, parents/carers join the online series	Medical review with psychiatrist/nurse practitioner and GP liaison and 6 × 60-min weekly online psychoeducation sessions on a continuous cycle (what is eating disorder, refeeding strategies, evidence-based treatments, practical nutrition, distress tolerance skills, broader support services (NGO partners attend session)	1:1 with psychiatrist registrar/nurse practitioner once a week for 45 min. Group-based session once a week for 60 min with psychiatrist, dietician, nurse, consumer, carer, social worker and/or psychologist	50 families completed the psychoeducation: One parent attended ≅ 80% sessions. Participants report increased confidence in supporting their child, clinicians report improved engagement with, and preparation for, treatment. The program is now embedded following assessment for all families engaging with the service	Engaging families challenging due to multiple commitments required, for example, 1:1, group sessions and GP appointments. Families keen to go straight into evidence-based treatment and perceive this program as a holding pattern
Online family-based treatment parent, a temporary COVID model (2020–21). The aim was to (1) provide education, support and intervention to parents awaiting allocation to FBT with an individual therapist; (2) activate peer support to maximise ‘buy in commitment and co-learning’; (3) provide FBT phase 1 principles, skills and interventions to parents; (4) maximise the YP’s private care team, for example, GP, private dietitian, or MH therapist	Families with a YP (0–18 years old within Western Metro Melbourne) assessed and diagnosed with AN or AAN until allocation to a MH therapist for individual FBT	1 × 30 min per group education session (Zoom) includes features and impact of AN and AAN, role of parents, multi-disciplinary care team, core phase 1 FBT tasks, GFBT format and structure. 6-week group (1–3 groups at the one time for 60 min on Zoom for 4–8 families): Brief family review (weight check, FBT phase 1 tasks), refeeding, emotions, parent–carer unity, relationship connection and trust, summary of key themes, learnings and actions	Group facilitators: Two mental health therapists (including psychologist registrar), peer support and education. Phone call support from therapist or nurse. Medical monitoring: acute, nurse/paediatrician; non-acute, GP. Where required – medical admission	179 families participated. Inpatient admissions: In 2021 47% increase in medical admissions compared to 2019 yet our length of stay reduced from 15 to 10.5 days. Outpatient: In 2022, N receiving outpatient mental health treatment increased by 88% compared to 2019. We provided a ‘good enough level of care’ for a much larger cohort. GFBT worked well for most but not all	Not always suitable for complex family presentations. Absence of engagement with the YP during GFBT. Expansion of care team, for example, GP, private therapist, CAMHS presented challenges in consistency in message and care team unity. Online forum diluted family commitment and engagement. Therapeutic alliance with families, for example, often different therapist for assessment, GFBT and individual FBT therapist. Online coordination presented multiple challenges

*Notes.* FBT = Family-Based Treatment; AN = anorexia nervosa; AAN = atypical anorexia nervosa; BN = bulimia nervosa; ARFID = avoidant restrictive food intake disorder; CAMHS = Child and adolescent mental health service; CYMHS = Child and Youth Mental Health Services; YP = young person; NGO = non-government organisation; GP = general practitioner; WFH = Work from Home; GFBT = group Family-Based Treatment.

*Principles of practice identified across service providers*: At the annual meeting of the 2022 Australia and New Zealand Academy of Eating Disorders (ANZAED) a workshop was conducted and facilitated by the authors with 87 registrants (13 from New Zealand), representing consumers and various disciplines across private practice, public service, and non-government organisations. Discussions were held in small groups focusing on the question: ‘What principles do we need to embrace moving forward with service provision?’

Five principles were endorsed. First, the importance of immediate availability of a low-resource intervention that was ‘good enough’. The experience of the pandemic suggests that immediate, time-limited online/telephone connection with parents/carers is appreciated by many families, even in the absence of a detailed, multi-disciplinary, face-to-face assessment. Such contact can lead to substantial improvements to those families who participate.^1^

Second, the necessity of providing hybrid services to the young person with an important non-negotiable of face-to-face meetings for regular medical monitoring. Given the immense burden anorexia nervosa imposes on families, we must carefully consider which elements of service delivery can be delivered face-to-face or remotely.

Third, the immediate importance of enhancing carer knowledge and skills. The usefulness of psychoeducation, presented in digestible units, and tailored to the circumstances and context of the family, was highly valued.

Fourth, utilisation of lived experience was especially important for increasing the acceptability and uptake of services, building hope in families, engaging families in treatment, and enabling a greater understanding of the skills needed to refeed one’s child.

Fifth, resources for evaluation of service delivery are essential for dissemination of what is working and what is not working so we can continue to respond flexibly in providing the best service delivery to a vulnerable client group.
